# The Next Volume

**Published:** 1867-11

**Authors:** 


### Editorial.

***The Next Volume.***

On and after the 1st January, ensuing, the JOURNAL will be issued semi-monthly, at the uniform rate of *Three Dollars* a year in advance, or *Four Dollars* after six months. Each No.

will contain thirty-two pages, thus adding sixteen pages to the month, or one hundred and ninety-two to the volume. Regular correspondents have been engaged in Paris, Vienna, New York, and Philadelphia, who will keep our readers "posted" on all matters of medical news and advance. The increased number of pages will afford space which has been sorely needed for abstracts of valuable articles as they appear in other medical periodicals, book notices, etc. A series of papers, prepared by the Editor, will give an outline of the present condition of General Pathology and Therapeutics. In this series, an effort will be made to condense the subject, so far as possible, to aphorisms.

A large number of valuable communications are now on file for publication, and will appear as space will permit. All matters of local professional interest will be duly chronicled.

Briefly, the Editor has made arrangements, whereby he will be enabled to devote a large share of his time to the preparation of a journal which he trusts will meet with the continued favor and confidence of the profession. For the large additions already made to the subscription list since his return to the editorial chair, and their numerous and valuable communications, he returns sincere thanks.

